# C-Type Lectin Receptor (CLR)–Fc Fusion Proteins As Tools to Screen for Novel CLR/Bacteria Interactions: An Exemplary Study on Preselected *Campylobacter jejuni* Isolates

**DOI:** 10.3389/fimmu.2018.00213

**Published:** 2018-02-13

**Authors:** Sabine Mayer, Rebecca Moeller, João T. Monteiro, Kerstin Ellrott, Christine Josenhans, Bernd Lepenies

**Affiliations:** ^1^Immunology Unit and Research Center for Emerging Infections and Zoonoses, University of Veterinary Medicine, Hannover, Germany; ^2^Medical School Hannover, Institute for Medical Microbiology, Hannover, Germany; ^3^German Center for Infection Research (DZIF), Partner Site Hannover–Braunschweig, Germany; ^4^Max von Pettenkofer Institute, Ludwig Maximilian University Munich, Munich, Germany; ^5^German Center for Infection Research (DZIF), Partner Site Munich, Germany

**Keywords:** C-type lectin receptors, *Campylobacter jejuni*, innate immunity, flow cytometry, confocal microscopy, ELISA assay, screening tools, Dectin-1 receptor

## Abstract

C-type lectin receptors (CLRs) are carbohydrate-binding receptors that recognize their ligands often in a Ca^2+^-dependent manner. Upon ligand binding, myeloid CLRs in innate immunity trigger or inhibit a variety of signaling pathways, thus initiating or modulating effector functions such as cytokine production, phagocytosis, and antigen presentation. CLRs bind to various pathogens, including viruses, fungi, parasites, and bacteria. The bacterium *Campylobacter jejuni* (*C. jejuni*) is a very frequent Gram-negative zoonotic pathogen of humans, causing severe intestinal symptoms. Interestingly, *C. jejuni* expresses several glycosylated surface structures, for example, the capsular polysaccharide (CPS), lipooligosaccharide (LOS), and envelope proteins. This “Methods” paper describes applications of CLR–Fc fusion proteins to screen for yet unknown CLR/bacteria interactions using *C. jejuni* as an example. ELISA-based detection of CLR/bacteria interactions allows a first prescreening that is further confirmed by flow cytometry-based binding analysis and visualized using confocal microscopy. By applying these methods, we identified Dectin-1 as a novel CLR recognizing two selected *C. jejuni* isolates with different LOS and CPS genotypes. In conclusion, the here-described applications of CLR–Fc fusion proteins represent useful methods to screen for and identify novel CLR/bacteria interactions.

## Introduction

C-type lectin receptors (CLRs) are pattern recognition receptors and are known to sense pathogen-associated molecular patterns as well as danger-associated molecules. Upon ligand recognition, CLRs trigger a variety of functions, including the production of inflammatory mediators, the phagocytosis of pathogens, or intracellular signaling (1, 2). The carbohydrate recognition domain (CRD) mediates the binding of CLRs to their specific ligands. One well-described example for a CLR–ligand pair is the CLR Mincle and its ligand trehalose-6,6'-dimycolate (TDM), a unique glycolipid present in the cell wall of mycobacteria (3, 4). Crystal structure analyses of the bovine (5) and human (6) Mincle CRD revealed that the two glucose moieties and one acyl chain of TDM and its synthetic analog trehalose-6,6'-dibehenate interact with Mincle. However, for the majority of CLRs, their glycan ligands and binding mode to their respective ligands are still incompletely understood.

The main function of CLRs is the recognition of highly conserved glycans and glycoproteins located on the surface of pathogens including viruses, parasites, fungi, and bacteria (3, 7–9). However, CLRs may also sense endogenous danger signals released by damaged and necrotic cells (10–13). Dectin-1 is a CLR that is predominantly expressed by monocytes, dendritic cells (DCs), and macrophages (14). It has been described to bind to β-1,3-glucans present in the cell wall of several fungal pathogens. Dectin-1 was shown to recognize various fungal pathogens such as *Pneumocystis carinii, Candida albicans, Aspergillus fumigatus*, and *Cryptococcus neoformans* (15).

Fc fusion proteins are established tools to identify novel receptor–ligand interactions. To date, CLR–Fc fusion proteins have been successfully used to screen for novel CLR/pathogen interactions, as demonstrated for fungal interactions, such as the recognition of *P. carinii* by Mincle (16). In addition, several previously unknown CLR/bacteria interactions were identified using CLR–Fc fusion proteins, including the Mincle/*Streptococcus pneumonia*e (*S. pneumoniae*) interaction (17) or the SIGNR3/*Lactobacillus acidophilus* interaction (18). Moreover, CLEC5A (MDL-1) was found to be an important receptor for *Listeria monocytogenes* that impacts macrophage and neutrophil functions in *Listeria*-induced innate immunity (19). Recently, CLR–Fc fusion proteins were used to identify novel CLRs that interact with mycobacteria. Here, CLEC9A was identified as a receptor that was crucial for the regulation of signal transduction and cytokine production during *Mycobacterium tuberculosis* infection (20). Besides the identification of pathogen-derived CLR ligands, CLR–Fc fusion proteins also allow to screen carbohydrate libraries for novel CLR ligands. Identified ligands can then be further evaluated for their utility to target CLRs on antigen-presenting cells (21–24). Indeed, the ligation of cell surface CLRs can induce various responses such as phagocytosis, cell adhesion, cytokine and chemokine release, as well as antigen presentation (25), rendering CLRs promising targets for immune modulation.

The enteropathogen *Campylobacter jejuni* (*C. jejuni*) expresses various virulence factors that allow for motility, adhesion, and invasion of host tissue, leading not only to acute self-limiting gastrointestinal illness but also to autoimmune disorders like Guillain–Barré syndrome (26, 27). Diarrheal *Campylobacter* species can colonize the intestines of many different host species, ranging from farm animals such as cattle and chicken to humans. Interestingly, they persistently colonize most nonhuman species without overt symptoms, verging on commensalism, while they cause acute intestinal disease in humans (28). *C. jejuni* is genetically quite variable and exists as generalist variants able to colonize various host species equally well (29, 30) and as specialist variants, which usually have only one preferred host species (31). In addition, each individual *C. jejuni* strain has the intrinsic property to vary its phenotype, for instance, by phase variation and contingency genes (32, 33). *Campylobacter* sp. are the only bacteria to date which express functional *N*- and *O*-glycosylation modules (34). Surface-exposed glycolipids such as the capsular polysaccharide (CPS) (35) and the lipooligosaccharide (LOS) (36) of the outer membrane play a pivotal role in host interaction and evasion by *C. jejuni*. In addition, *C. jejuni* expresses several cell-envelope-located *O*- and *N*-linked glycoproteins (37, 38). Recent studies identified CLRs that are involved in *C. jejuni* recognition (39, 40). In one study, hMGL–Fc was shown to interact with *C. jejuni*-derived glycoproteins (39). Another study used a murine CLR–hFc fusion protein library and showed LMIR5 to bind to *C. jejuni* (40). These studies point to a role of CLRs in host interplay and modulating the host immune response against *C. jejuni*.

This “Methods” paper presents a combination of innovative techniques to screen for and study CLR/bacteria interactions, using *C. jejuni* as a representative example. All applied methods are based on CLR–hFc fusion proteins in which the extracellular part of the respective murine (m) or human (h)CLR containing the CRD has been fused to the Fc fragment of human IgG_1_ molecules, thus leading to dimer formation. ELISA-based methods allow for a high-throughput prescreening for potential CLR interactions with bacteria, followed by flow-cytometric analyses of identified candidates as a confirmatory method. To visualize and confirm binding of CLRs to bacteria *in situ*, confocal microscopy can be applied and was used in this study to visualize the binding of Dectin-1 to *C. jejuni*.

## Materials and Methods

### *C. jejuni* Strains, Culture Conditions, and Preparation of Bacteria for Interaction Studies

*Campylobacter jejuni* strains used were from two strain collections (29, 41) assembled in Germany between 2011 and 2016. We selected two different, but related, generalist strains that are from two frequent *C. jejuni* lineages that can colonize well with various animal species including humans and that cause frequent diarrheal diseases in the latter. Strain MHH-24 is equivalent to isolate FBI-Zoo 06025 (ST22) from Ref. (29) and was isolated from raw milk (cattle), and strain MHH-19, a human enteritis isolate, is equivalent to isolate FBI-Zoo 07079 (ST19) from the more recent case–control study (41). MHH-19 has well-recognized genetic determinants for capsule and LOS types (own unpublished data); however, strain MHH-24 has not been typeable so far by molecular methods for LOS or CPS (own unpublished data). Both strains’ LOS and CPS glycans or other surface glycans have not been characterized biochemically so far. For the plate assay and FACS-based assay, heat-fixed bacteria were used, which allow for better staining of the bacteria with the fluorescent Syto61 dye (Thermo Scientific), while for immunofluorescent individual bacterial labeling, PFA-fixed bacteria were prepared, which permit a superior surface preservation of the cells and better storage capacity. Bacteria were grown on blood agar plates (Columbia agar, supplemented with 5% sheep blood, Oxoid, Germany) and diluted in sterile 1x PBS at an OD_600_ of 1. Heat fixation was performed at 65°C in a heating block for 5 h. Heat-inactivated bacteria were stored at 4°C for a maximum of 3 weeks. Fresh 2% PFA as an alternative fixing agent for immunofluorescent labeling was prepared in 100 mM sterile-filtered potassium phosphate buffer, pH = 7.0, and bacteria were fixed twice for 1 h at room temperature (RT), with centrifugation (6.000 × *g*, 10 min, RT) and one change of fixing agent in between. Afterward, the bacteria were centrifuged again and resuspended in a sterile solution of 0.1% glycine in PBS to quench the fixing agent, which prevents nonspecific attachment of proteins or cell clumping. Ultimately, the bacteria were resuspended in pure, sterile 1× PBS (pH = 7.4) after a final centrifugation step and stored at 4°C until further use, with a high storage capacity of several months.

### Generation of CLR–hFc Fusion Proteins

The production of the CLR–hFc fusion proteins was performed as previously described (23). Briefly, RNA was isolated from murine spleen and reverse-transcribed into cDNA using a reverse transcriptase (New England Biolabs, Ipswich, MA, USA). Polymerase chain reaction was applied to amplify the cDNA encoding the extracellular part of each CLR using specific primers (Table 1). The respective cDNA fragments were ligated into a pFuse-hIgG1-Fc expression vector (InvivoGen, San Diego, CA, USA). Next, CHO-S cells were transiently transfected with the vector construct using MAX reagent (InvivoGen). CLR–hFc fusion proteins were purified after 4 days of transfection from the cell supernatant using HiTrap protein G HP columns (GE Healthcare, Piscataway, NJ, USA). To confirm the purity of each CLR–hFc fusion protein, the protein was analyzed by dodecyl sulfate polyacrylamide gel electrophoresis (SDS-PAGE) and subsequent Coomassie staining as well as Western blot using an anti-human IgG- horseradish peroxidase (HRP) antibody (Dianova, Hamburg, Germany).

**Table 1:** DNA sequences of primers used for amplification of the extracellular domain of the respective CLRs.

CLR	Primer
mCLEC12A	FW 5′- GAATTCTTTGGCAACAGAAATGATAA-3′
	RV 5′- AGATCTGCCATTCAACACACTTTCCA-3′
mDectin-1	FW 5′- GAATTCTTCAGGGAGAAATCCAGAGG-3′
	RV 5′- AGATCTTGAAGAAGTATTGCAGATTTGGTT-3′
mDectin-2	FW 5′- CCATGGAGAAAACATCATTCCAGCCCC-3′
	FW 5′- GAATTCCTGGAGCACCAGTGAGCAGAAC-3′
mCLEC9a	FW 5′-GAATTCGGGCATCAAGTTCTTCCAGGTATCC-3′
	RV 5′-CCATGGTGCAGGATCCAAATGCCTTCTTC-3′
mDCAR	FW 5′- CCATGGAACTTGACAGGTACCATTCATT-3′
	RV 5′- AGATCTTAAGTTTATTTTCTTCATCTGAC-3′
mSIGNR3	FW 5′- GAATTCCATGCAACTGAAGGCTGAAG-3′
	RV 5′- AGATCTTTTGGTGGTGCATGATGAGG-3′
mMGL-1	FW 5′- CCAGTTAAGGAGGGACCTAGGCAC-3′
	RV 5′- AGCTCTCCTTGGCCAGCTTCATC-3′
mMDL-1	FW 5′- GAATTCCCCCACGGAGAGCTACGGAACCA-3′
	RV 5′- CCATGGTGGCATTCATTTCGCAGATCCA-3′
hDC-SIGN	FW 5′- GAATTCCATGCAACTGAAGGCTGAAG-3′
	RV 5′- GATCTTTTGGTGGTGCATGATGAGG-3′
hL-SIGN	FW 5′- GAATTCCTATCAAGAACTGACCGATTTG-3′
	RV 5′- CCATGGATTCGTCTCTGAAGCAGGC-3′

### Western Blot

After protein separation using SDS-PAGE, the proteins were transferred to a nitrocellulose membrane for 1 h at 5 V. The membrane was blocked for 1 h with 5% milk powder in TBS and 0.1% Tween-20 (TBS-T) followed by a 1-h incubation with an anti-human IgG antibody conjugated to HRP (Dianova). The membrane was washed three times with TBS-T, for 5 min each. Detection of the CLR–hFc fusion proteins was performed using the Amersham ECL Western blotting detection reagent (GE Healthcare).

### ELISA-Based Binding Studies

A half-area microplate (Greiner Bio-One GmbH, Frickenhausen, Germany) was coated with 3 × 10^8^ CFU/ml heat-inactivated *C. jejuni* for 3.5 h at RT. Non-adherent bacteria were washed away, and the plate was blocked with buffer containing 1% BSA (Thermo Fisher Scientific/Invitrogen, Darmstadt, Germany) in 1x PBS for 2 h at RT. After washing the wells, 200 ng of each respective CLR–hFc fusion protein in lectin-binding buffer (50 mM HEPES, 5 mM MgCl_2_, and 5 mM CaCl_2_) was added to the bacteria and incubated for 1 h at RT. Then, a 1:5.000-diluted HRP-conjugated goat anti-human IgG antibody (Dianova) was added for 1 h at RT. Finally, the substrate solution [*o*-phenylenediamine dihydrochloride substrate tablet (Thermo Fisher Scientific), 24 mM citrate buffer, 0.04% H_2_O_2_, 50 mM phosphate buffer in H_2_O] was added to the samples, and the reaction was stopped with 2.0 M sulfuric acid. Data was collected using a Multiskan Go microplate spectrophotometer (Thermo Fisher Scientific) at a wavelength of 495 nm. Four independent experiments were performed with technical triplicates each.

### Flow Cytometry-Based Binding Studies

To detect the bacteria and exclude them from debris, 3–6 × 10^7 ^CFU/ml heat-inactivated *C. jejuni* were stained with 1 µM of the DNA-staining dye Syto61 (Thermo Fisher Scientific) and incubated for 30 min at RT. Subsequently, samples were incubated for 1 h with 200 ng of the respective CLR–hFc fusion protein in lectin-binding buffer. After washing once with lectin-binding buffer, the bacterial pellet was stained with a PE-conjugated goat anti-human Fc (Dianova) antibody solution and incubated for 25 min at 4°C. Finally, flow-cytometric analysis was performed using an Attune NxT Flow Cytometer (Thermo Fisher Scientific). Data analysis was performed using the FlowJo Software (FlowJo, Ashland, OR, USA). As a control, hFc protein was used to exclude the nonspecific binding of *C. jejuni* to the Fc part of the CLR–Fc fusion proteins. Besides the use of the hFc protein, the secondary antibody alone served as an additional negative control. Three independent experiments were done with technical duplicates each.

### Confocal Fluorescence Microscopy-Based Binding Studies

Cover slides (Thermo Fisher Scientific) were cleaned with 70% ethanol and coated with poly-L-lysine solution (Sigma-Aldrich, St. Louis, MO, USA) for 30 min at 60°C. 6 × 10^7^ CFU/ml *C. jejuni* isolate MHH-19 fixed with 2% PFA was washed with 1x PBS and incubated overnight (o.n.) with 500 ng Dectin-1–hFc and hFc in lectin-binding buffer at 4°C. After washing two times with lectin-binding buffer, samples were incubated for 2 h with 1:200-diluted goat anti-human Fc Alexa Fluor (AF) 488-conjugated antibody (Dianova) at 4°C. Next, samples were washed with 1x PBS, applied onto poly-L-lysine-coated cover slides, and incubated for 45 min at 37°C. In addition, a sample with *C. jejuni* and the secondary antibody only was used as a negative control. Finally, the cover slides were mounted on microscopic slides (Roth, Karlsruhe, Germany) with proLong™ gold antifade mountant containing DAPI (Thermo Fisher Scientific), sealed and visualized using a TCS SP5 confocal inverted-base fluorescence microscope (Leica, Nussloch, Germany) equipped with a HCX PL APO 63 × 1.4 oil immersion objective. To avoid the detection of artifacts, PFA-fixed bacterial samples were inspected visually by a high-magnification microscopy (100× lens magnification) for clumps before performing hFc fusion protein co-incubation. Only bacterial preparations without any visible clumps were further used. Three independent experiments were performed, each with three randomly selected pictures.

### Statistical Analysis

All data are presented as mean ± SD. Unpaired, one-tailed Student’s *t*-test was applied to determine the significance between CLR candidates and the hFc control. Data were analyzed using the GraphPad Prism software (version 7.02).

## Results

### Generation and Detection of CLR–hFc Fusion Proteins Used in This Study

**Figure 1:**
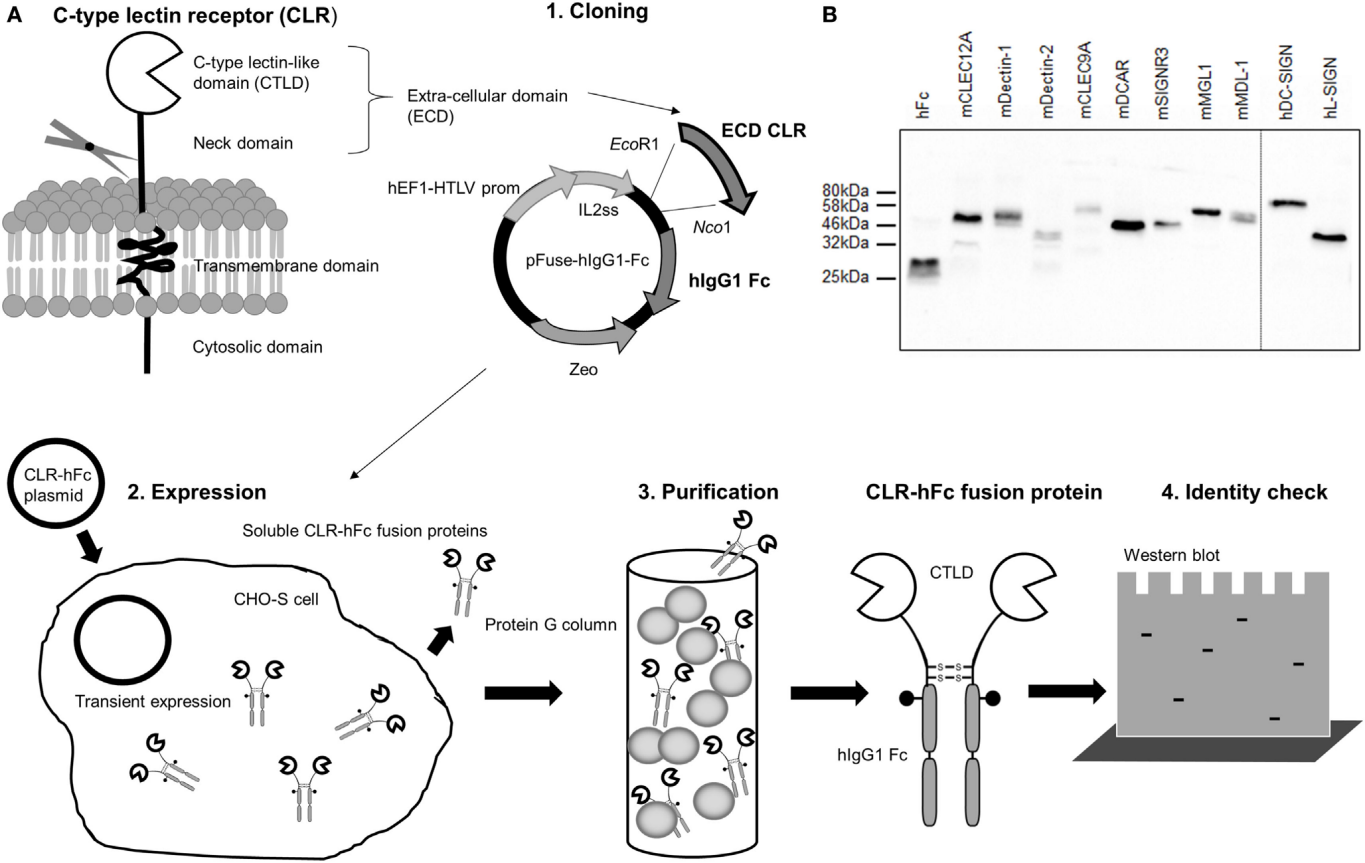
**(A)** Overview of the generation and production of C-type lectin receptor (CLR)–hFc fusion proteins. (1) First, the cDNA encoding for the carbohydrate recognition domain of the respective CLR is cloned into the expression vector and thereby fused to the cDNA fragment encoding for human IgG1-Fc. (2) CHO-S cells are transiently transfected with the expression vector construct, and the respective soluble CLR–hFc fusion proteins are secreted. (3) The cell supernatant is purified, and (4) the presence of the CLR–Fc fusion proteins is detected. **(B)** Western blot of selected CLR–hFc fusion proteins. To confirm the successful CLR–hFc fusion protein production, supernatants of transiently transfected cells were purified using protein G columns, and detection by Western blot was performed using a horseradish peroxidase-conjugated anti-hFc antibody.

The generation of CLR–hFc fusion proteins required several steps (Figure 1A, 1–4). The first step was the cloning of the cDNA fragment encoding for the extracellular part of each CLR (containing the CRD) and its fusion to the Fc fragment of human IgG1 in the pFuse-hIgG1-Fc expression vector (1). Next, mammalian CHO-S cells were transfected with this vector construct (2). The use of a mammalian cell line such as CHO-S cells ensured that soluble CLR–hFc fusion proteins were secreted into the supernatant that carried mammalian-type glycosylation. Finally, the supernatant was harvested, followed by purification of the respective fusion proteins using protein G columns (3). To confirm the presence and purity of the respective CLR–hFc fusion proteins after purification, SDS-PAGE and subsequent Coomassie staining and a Western blot were performed (Figure 1B). Bands at the expected size of the respective CLR–hFc fusion protein showed the presence of each CLR–hFc fusion protein.

### Prescreening Using an ELISA-Based Assay

**Figure 2:**
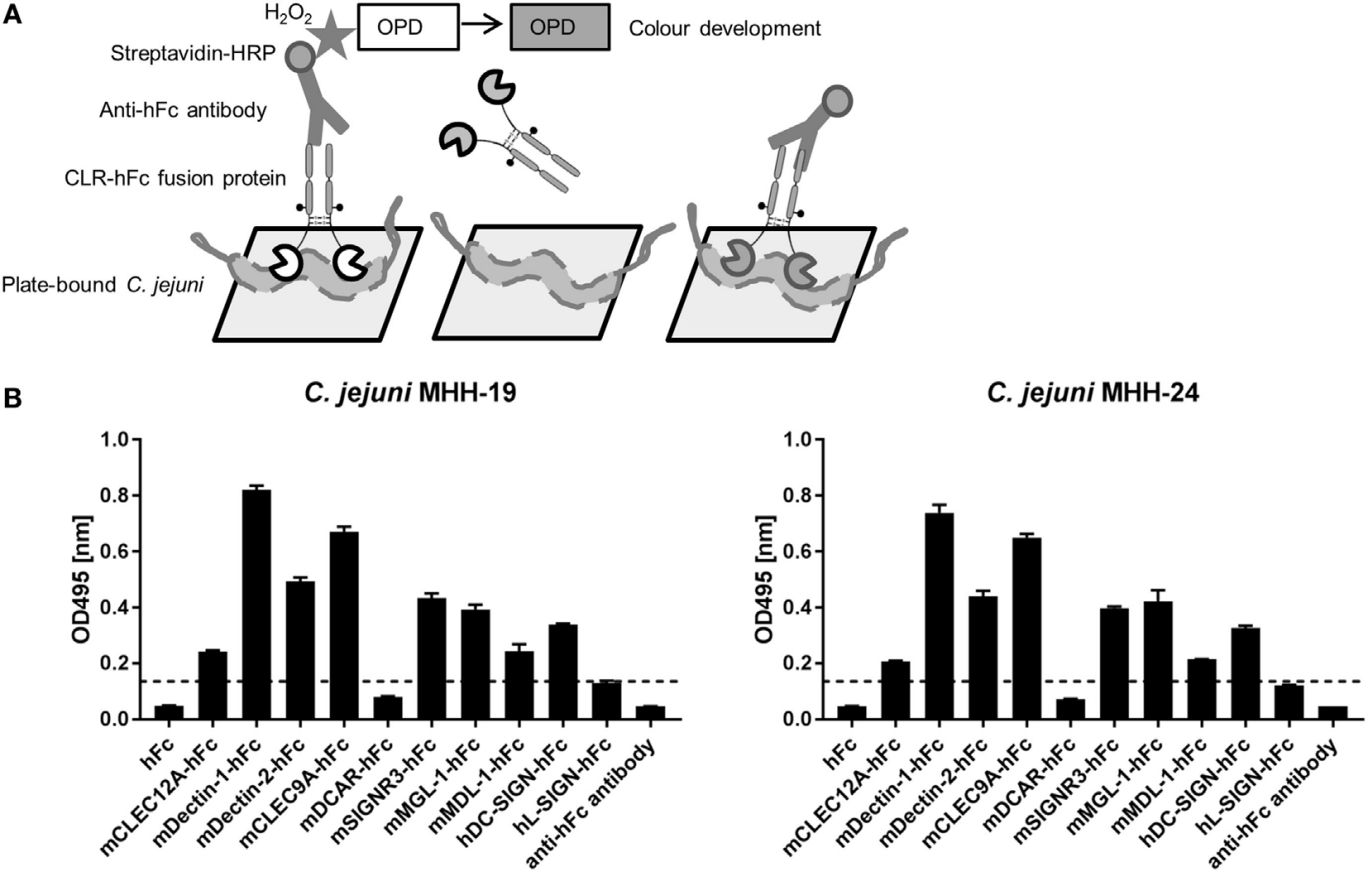
**(A)** Schematic representation of the ELISA-based-binding study using the C-type lectin receptor (CLR)–hFc fusion proteins. Immobilized *C. jejuni* was incubated with the respective CLR–hFc fusion proteins. The detection of bound fusion proteins was performed using a horseradish peroxidase (HRP)-conjugated anti-hFc antibody and subsequent colorimetric detection (details are given in Section “Materials and Methods”). **(B)** Several CLR–hFc fusion proteins were analyzed for their interaction with heat-inactivated immobilized *C. jejuni*. This prescreening showed no or only marginal binding of DCAR–hFc and L-SIGN–hFc. The CLR–hFc fusion proteins CLEC9A, DC-SIGN, Dectin-1, Dectin-2, MDL-1, MGL-1, CLEC12A, and SIGNR3 exhibited weak to marked binding to both *C. jejuni* isolates. Representative data from one out of four independent experiments are shown (technical triplicates for each condition). The dashed line represents the defined threshold for CLR/bacteria interactions in the ELISA-based assay. Student’s *t*-test was performed to compare all CLR–hFc fusion proteins with absorbance above the threshold to the hFc control alone. For both *C. jejuni* isolates, a highly significant binding (****p ≤ 0.0001) was observed for the CLR–hFc fusion proteins mCLEC12A–hFc, mDectin-1–hFc, mDectin-2–hFc, mCLEC9A–hFC, mSIGNR3–hFC, mMGL-1–hFc, mMDL-1–hFc, and hDC-SIGN–hFc.

In a first screening, the binding of the CLR–hFc fusion proteins to two different *C. jejuni* isolates (MHH-19 and MHH-24) was tested using an ELISA-based method. After immobilization of the heat-inactivated bacteria on the ELISA plate and incubation with the respective CLR–hFc fusion proteins, their interaction with the *C. jejuni* isolates was determined by colorimetric detection (Figure 2A). Several controls were included, such as the incubation of the CLR–hFc fusion proteins on non-coated wells (data not shown) or with the hFc protein alone to exclude unspecific binding of the Fc fragment to *C. jejuni*. In general, the CLR–hFc fusion proteins exhibited a similar binding pattern to both *C. jejuni* isolates (Figure 2B). No binding was observed for DCAR–hFc and L-SIGN–hFc. All other CLR–hFc fusion proteins displayed weak to strong binding to *C. jejuni* in the ELISA-based assay and were considered as potential receptors for the *C. jejuni* isolates MHH-19 and MHH-24.

### Confirmatory Test Using a Flow Cytometry-Based Assay

**Figure 3:**
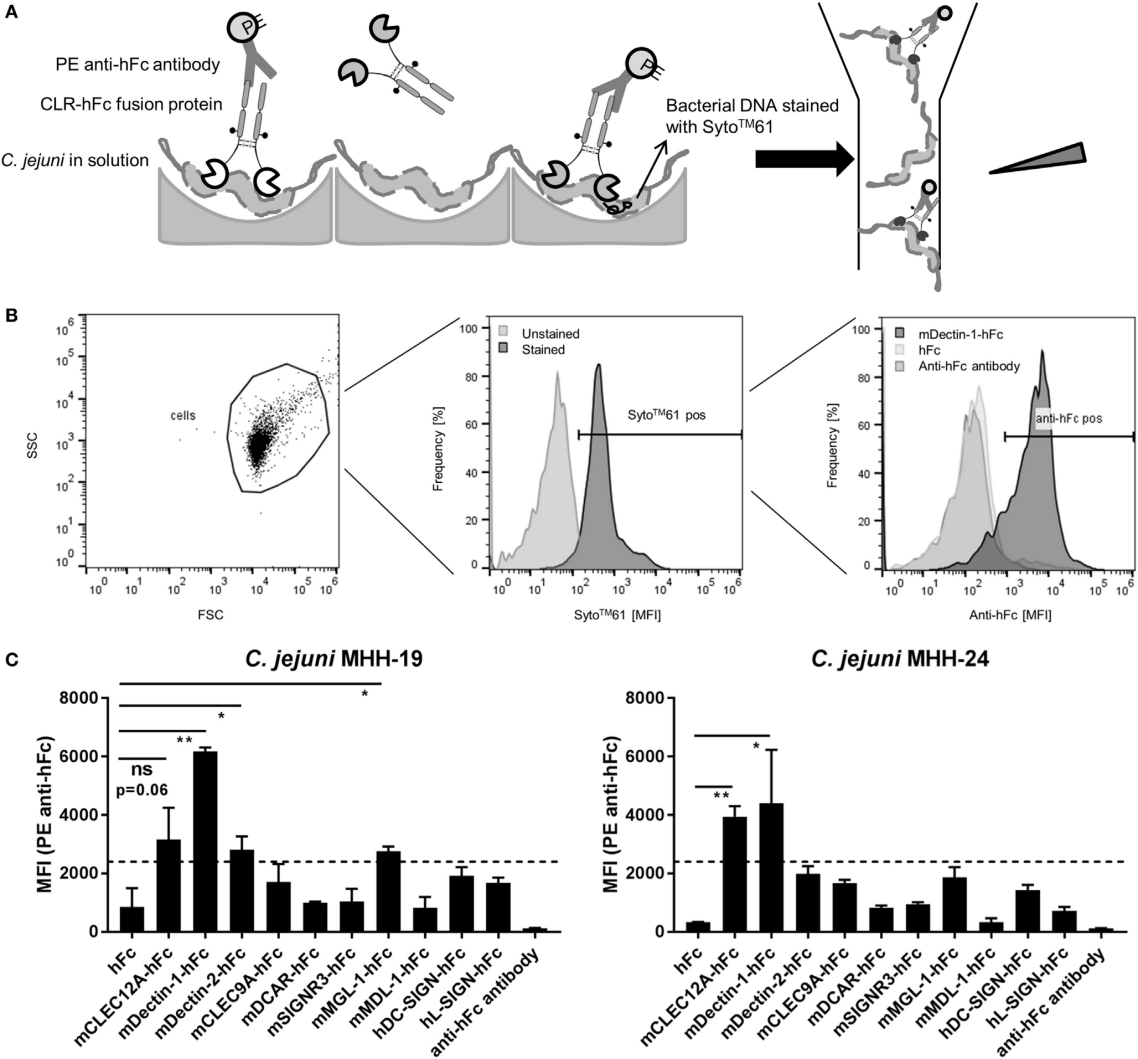
**(A)** Overview of the flow cytometry-based protocol to detect bacterial recognition by C-type lectin receptor (CLR)–hFc fusion proteins. *C. jejuni* was incubated with the respective CLR–hFc fusion proteins in solution. Binding was detected using an anti-hFc antibody. Subsequently, samples were analyzed by flow cytometry based on their morphological and fluorescence properties. **(B)** Histoplots show the gating strategy to detect the binding of CLR–hFc fusion proteins to *C. jejuni* in a representative experiment. Cells were discriminated from debris by gating on forward scatter (FSC) and side scatter (SSC). *C. jejuni* was further gated on DNA staining, indicated by Syto61 positivity (pos.). Finally, Syto61-positive bacteria were analyzed for Dectin-1–hFc binding. hFc and the secondary antibody alone were used as controls. **(C)** Representative data from one out of three independent experiments of the flow cytometry-based CLR–hFc-binding studies to the *C. jejuni* isolates MHH-19 and MHH-24 (technical duplicates each). Similar binding profiles for both isolates were observed. While Dectin-1–hFc and CLEC12A–hFc exhibited binding to *C. jejuni*, all other tested CLR–Fc fusion proteins displayed marginal to no binding. The dashed line represents the defined threshold for CLR/bacteria interactions in the flow cytometry-based assay. Data are expressed as mean ± SD. Student’s *t*-test was performed to compare the binding of CLR–Fc fusion proteins above the threshold to hFc alone. For all statistical analyses, *p*-values of <0.05 were considered to be significant: ns = not significant, *p ≤ 0.05, **p ≤ 0.01.

To verify and extend the results from the ELISA-based detection method, a flow cytometry-based protocol to screen for CLR/bacteria interactions in solution was established. To this end, *C. jejuni* was incubated with CLR–hFc fusion proteins, and subsequent binding was detected upon staining with a PE-conjugated anti-hFc antibody (Figure 3A). The gating strategy is displayed in Figure 3B and is based on the gating of bacteria in the forward-scatter/side-scatter plot, followed by gating on Syto61-positive events. Incubation with the Dectin-1–hFc fusion protein led to a marked shift in the fluorescence intensity, indicating the binding of Dectin-1–hFc to *C. jejuni*. For both *C. jejuni* isolates, no binding was observed for staining with the hFc fragment or with the secondary antibody alone. The lack of binding of the hFc fragment to *C. jejuni* illustrates the specificity of the recognition of the *C. jejuni* isolates by Dectin-1–hFc. The analysis of the binding studies shows that both tested *C. jejuni* isolates were significantly recognized by Dectin-1–hFc and, to a lesser extent, by CLEC12A–hFc (Figure 3C). All other CLR–hFc fusion proteins included in the flow cytometry-based assay exhibited no or only marginal binding to both *C. jejuni* isolates. Interestingly, no binding of CLEC9A–hFc to both *C. jejuni* isolates was detected using the flow cytometry-based assay. This finding may either suggest a false-positive result in the ELISA-based assay or may be due to internal *C. jejuni* ligands that are not accessible in the flow cytometry-based assay. In summary, the CLR–hFc fusion proteins exhibited a similar binding profile to both *C. jejuni* isolates in the flow cytometry-based assays and revealed Dectin-1 as a novel candidate receptor for *C. jejuni* recognition.

### Visualization of CLR/Bacteria Interactions Using Confocal Microscopy

**Figure 4:**
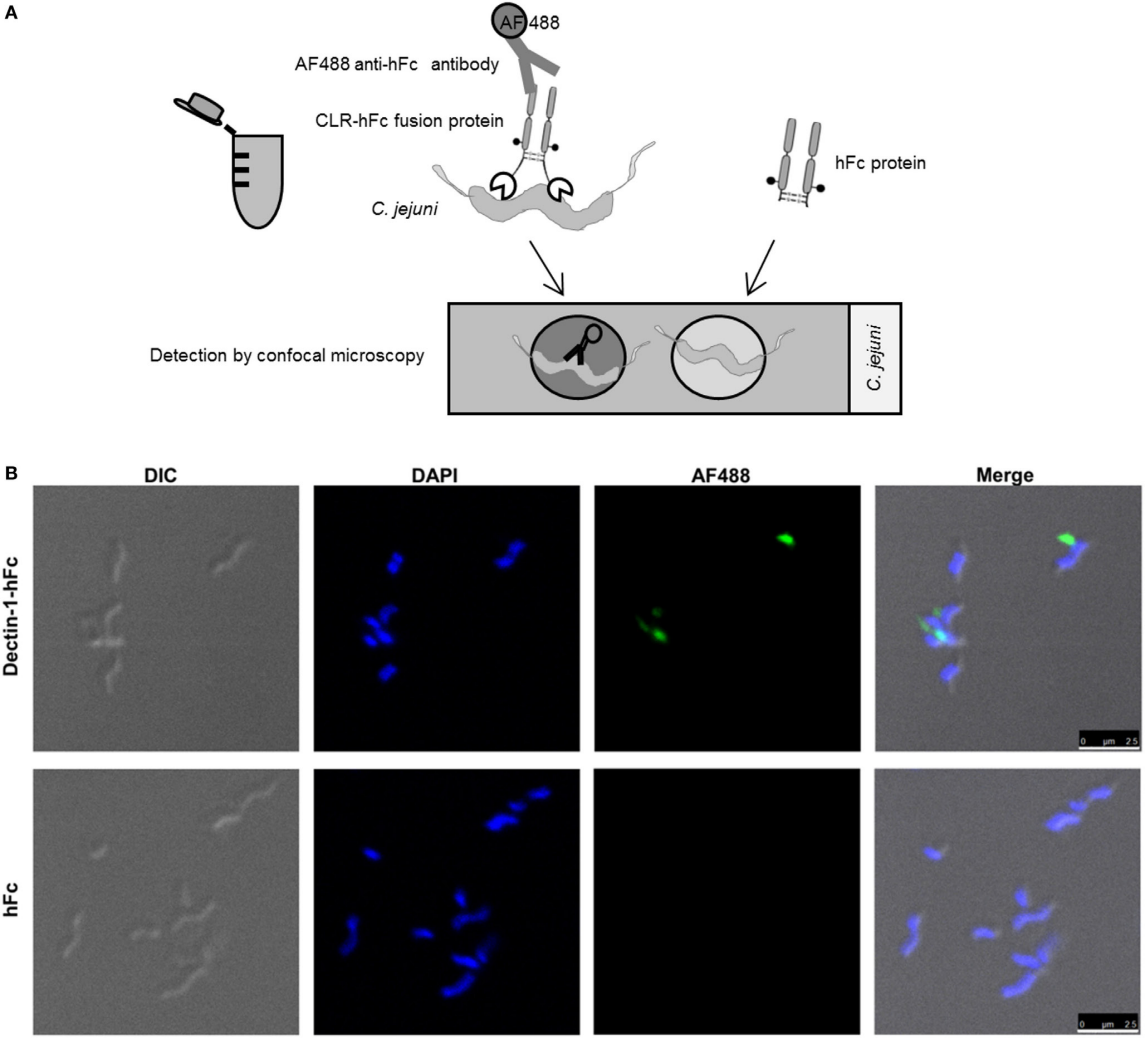
**(A)** Schematic representation of the C-type lectin receptor (CLR)–hFc fusion protein-binding studies using confocal microscopy. Bacteria were incubated with the CLR–hFc fusion proteins. Bound CLR–hFc fusion proteins were detected by an Alexa Fluor (AF) 488-conjugated anti-hFc antibody. To exclude unspecific binding, bacteria were also incubated with hFc as control protein or with the secondary antibody alone. Bacteria were transferred onto cover slips, mounted onto microscopic slides, and visualized using confocal microscopy. **(B)** Bacteria were incubated with Dectin-1–hFc (upper panels) or hFc protein (negative control, lower panels). Differential interference contrast (DIC) was included to visualize the bacterial cell wall. Bacterial DNA was stained with DAPI (shown in blue). The binding of the Dectin-1–Fc fusion protein to *C. jejuni* was detected using an AF488-conjugated secondary antibody (shown in green). While Dectin-1–hFc recognizes *C. jejuni*, the hFc control and the secondary antibody alone showed no signal in the AF488 channel. The experiment was repeated three times with three randomly selected pictures each, and similar results were revealed. Scale bar indicates 2.5 µm.

To visualize the identified Dectin-1–hFc interaction with *C. jejuni in situ*, confocal microscopy after immunofluorescent labeling was applied. The incubation of *C. jejuni* with Dectin-1–hFc and hFc control protein was performed in solution. Subsequently, bacteria were immobilized on poly-L-lysine-coated cover slides, and individual CLR/*C. jejuni* interactions were visualized using a confocal laser-scanning microscope (Figure 4A). The results confirmed Dectin-1 binding to *C. jejuni*, whereas no binding was observed for hFc (Figure 4B) and the secondary antibody alone (data not shown). Furthermore, merging the fluorescence channels and the differential interference contrast showed that the Dectin-1–hFc signal colocalized with patches on the bacterial cell periphery. This finding suggests that Dectin-1–hFc recognizes a cell-envelope component of *C. jejuni*. In conclusion, the combination of ELISA-, flow cytometry-, and confocal microscopy-based methods highlights the utility of CLR–Fc fusion proteins to identify novel CLR/bacteria interactions as demonstrated here using *C. jejuni* as an example. The functional role of the *C. jejuni* recognition by Dectin-1 can now be further elucidated in future studies.

## Discussion

This article presents three distinct methods to detect and verify novel CLR/bacteria interactions. Often, the identification of novel CLR/pathogen interactions is the first step to unravel the interplay of the host innate immune system with bacterial pathogens. Each of the methods can be applied for different purposes and has certain advantages and drawbacks (presented in Table 2). The ELISA-based method allows for a high-throughput screening of bacteria collections. Due to the possibility of false-positive results caused by protein aggregation on the ELISA plate, this method is mainly suitable for an initial prescreening using the whole CLR–hFc fusion protein library and requires confirmation by additional methods. To confirm initially identified CLR/bacteria interactions, flow-cytometric analysis represents a useful method that has several advantages. First, binding takes place in solution, thus avoiding protein aggregation on the ELISA plate. Second, the flow cytometry-based method offers the possibility to discriminate between bacteria and debris using an appropriate gating strategy. Third, it allows for narrowing down the localization of ligands to the bacterial surface, whereas the ELISA-based method may lead to a partial lysis of bacteria, thus releasing internal ligands. By contrast, confocal (fluorescence) microscopy offers the opportunity of visualizing CLR/bacteria interactions for single-bacteria *in situ*, thus enabling colocalization studies to further characterize the bacterial ligand. All described methods can be easily applied to other Gram-positive and Gram-negative bacterial species.

**Table 2:** Advantages and drawbacks of the ELISA-based, flow cytometry-based, and confocal microscopy-based methods to detect novel CLR/bacteria interactions.

Method	Advantages	Drawbacks	Main purpose
ELISA	-High-throughput screening possible -Fast screening	-False-positive results possible due to protein aggregation on the plate -Requires pure pathogen samples	Prescreening for CLR/bacteria interactions

Flow cytometry	-Semi-quantitative comparisons possible -Exclusion of debris due to appropriate gating -Information on a large number of cells for statistical analyses	-Restricted to detectable events in SSC and FSC -Limited to ligands present on the surface of pathogens (can also be an advantage)	Confirmation of CLR/bacteria interactions

Microscopy	-Colocalization studies possible -Visualization of CLR interactions with single bacteria (detection of intra-strain variation) -Extracellular and intracellular staining possible (preserved structure)	-Time-consuming -Requires advanced staining protocols	Direct visualization of CLR/bacteria interactions

*Campylobacter jejuni* is an interesting candidate for screening glycan-binding factors, since the bacteria are heavily glycosylated with various different glycan species and possess active genes for variable *O*- and *N*-glycosylation, providing abilities to glycosylate capsule, LOS, and proteins (34). In addition, strain-specific differences between various *C. jejuni* strains exist concerning surface determinants and glycosylation (42), based, for instance, on strain-specific genetic differences and phase variation (32). Even in each individual *C. jejuni* strain, a high-variation potential of the bacterial surface phenotype, for instance, LOS, capsule, or additional LOS glycosylation, exists (33, 43–45), which provides an interesting field of future study. Applying the CLR–hFc fusion protein library to screen for binding to preselected, molecularly typed *C. jejuni* isolates from two frequent generalist lineages, we identified Dectin-1–hFc as a promising candidate receptor for *C. jejuni*. Phongsisay et al. screened *C. jejuni* lysates using a murine CLR–hFc fusion protein library (40). In this previous study, the murine CLR LMIR5 was described to interact with *C. jejuni*, whereas other tested CLR/*C. jejuni* interactions remained negative. In bacterial lysates, components such as glycolipids are released and better accessible to potential receptors as in intact live or heat-inactivated bacteria. Thus, the CLR/*C. jejuni* interactions identified in the respective study may also include internal ligands that are not detected when intact bacteria are immobilized on the plate for ELISA-based detection or used in solution for the flow cytometry-based assay. In addition, the use of different *C. jejuni* strains or growth under different culture conditions may impact the recognition by CLRs. Since the surface interaction of the bacteria with host lectins might be more relevant for the colonization and infection process *in vivo*, we employed heat-inactivated intact bacterial cells instead of bacterial lysates for our screening procedures. In our present study, both isolates used in our study, which are genetically related, but not identical, exhibited a similar CLR-binding profile. It will be interesting to compare more *C. jejuni* strains including distantly related isolates, generalists, and specialists (30, 31) for lectin binding. In addition, phase variation, which is a common genetic mechanism used by *C. jejuni* to modulate its surface properties (32), may play a role in CLR recognition. Indeed, phase variation between strains and within the population of one *C. jejuni* isolate (33) may affect cell wall components, as has already been shown for the glycosylation of LOS (43), capsule (44, 45), and for other bacterial properties (32). In this context, it is worth noting that in our confocal microscopy *in situ* approach, bacterial cells showed an individual variation in Dectin-1 binding or the absence of binding. Phenotypical variation of this trait within the bacterial population has not been revealed in any earlier study on *C. jejuni* and might be explained by single-cell variation of a Dectin-1-binding surface determinant. This presents a very interesting opportunity for further study of individual bacterial intra-strain variation. In an earlier study, human MGL was shown to recognize *C. jejuni* through binding to *C. jejuni*-derived *N*-glycosylated proteins (39). For murine MGL-1, we observed only weak binding to *C. jejuni*, which may be due to experimental or strain differences, or to the different binding profiles between murine and human MGL isoforms. While two different orthologs (mMGL-1 and mMGL-2) are found in mice, humans only express one MGL isoform (hMGL). It is known that mMGL-2 displays a similar binding profile as hMGL which may account for the marginal binding observed for the mMGL-1 ortholog in our study (46, 47).

In this study, we have identified mouse Dectin-1 as a candidate receptor for the innate recognition of *C. jejuni*. To date, Dectin-1 has mainly been described as CLR-recognizing fungal pathogens. For instance, Dectin-1 binds to *C. albicans, A. fumigatus*, and *C. neoformans* (15). The Dectin-1 ligand recognized in the context of fungal infection is β-1,3-glucan (48), present in the cell wall of several fungi. Nevertheless, also parasites such as *Leishmania infantum* (49, 50) and *P. carinii* (51) were described to be sensed by Dectin-1. Interestingly, also an interaction of Dectin-1 in cooperation with TLR2 was shown for several *Mycobacterium* species (52). In *C. jejuni*, α-1,4-glucan has been reported as a capsule component (53).

The identification of candidate CLRs that play a role in bacterial recognition presents the first step to identify a distinct bacterial ligand for the respective receptor and may help to understand the interaction of bacteria with the host innate immune system. Identified CLR candidates can be further investigated for their relevance *in vitro* and *in vivo*. Using a comprehensive CLR–hFc library, Rabes et al. demonstrated that Mincle recognizes *S. pneumoniae* in a Ca^2+^-dependent manner (17). This work was extended by a recent study showing that Mincle recognizes *S. pneumoniae*-derived glucosyl-diacylglycerol in a serotype-specific fashion (54). To date, several CLR ligands have been identified by the use of CLR–Fc fusion proteins. For instance, one study revealed Mincle–hFc as a receptor sensing mannose and glucose-rich glycolipids extracted from *Malassezia pachydermatis* (55). In addition, the identification of distinct glycan ligands of CLRs offers the possibility for glycan-based CLR targeting to deliver vaccine antigens into antigen-presenting cells and to induce subsequent adaptive immune responses (22, 56–58). This approach has already been applied successfully to vaccine design using carbohydrate-based adjuvants (22, 59, 60). Besides CLR–Fc fusion protein libraries, reporter cell lines expressing the respective CLR are used to identify novel CLR–pathogen interactions and CLR ligands (61, 62). In addition, such reporter cell lines also allow for investigating if the identified CLR ligands act as potential agonists or antagonists. In conclusion, this “Methods” paper combines three different screening and confirmatory methods for the detection of CLR–hFc fusion protein binding by pathogens. It also highlights the utility of CLR–hFc fusion proteins to screen for novel CLR/bacteria interactions as a first step toward the identification of distinct bacterial CLR ligands and characterization of their biological functions.

## Author Contributions

SM, CJ and BL designed the research; SM and RM performed the research; JM and KE contributed to new reagents/analytical tools; SM, RM, CJ, and BL analyzed data; and SM, CJ, and BL wrote the paper with the help of the other authors.

## Conflict of Interest Statement

The authors declare that the research was conducted in the absence of any commercial or financial relationships that could be construed as a potential conflict of interest.
